# Emphasizing the Pharmacological Potentials of the Methanolic Extract of *Wedelia montana*: A Prominent Source of Veterinary Medicine

**DOI:** 10.1155/vmi/1914665

**Published:** 2026-01-28

**Authors:** Millat Hossain Mesu, Md Ashraf Uddin Chowdhury, Mohammad Arman, Israt Jahan, Sourav Kumar Shill, Md. Al Mamun, Md. Jahirul Islam Mamun, Md. Abdul Alim, Md. Tanvir Chowdhury, S. M. Moazzem Hossen

**Affiliations:** ^1^ Department of Pharmacy, International Islamic University Chittagong, Chattogram, 4318, Bangladesh, iiuc.ac.bd; ^2^ Department of Pharmacy, BGC Trust University Bangladesh, Chattogram, 4381, Bangladesh, bgctub-edu.com; ^3^ Department of Pharmacy, University of Asia Pacific, Dhaka, Bangladesh, uap-bd.edu; ^4^ Department of Pharmacy, Faculty of Biological Sciences, University of Chittagong, Chattogram, 4331, Bangladesh, cu.ac.bd

**Keywords:** analgesics, anthelmintic, antidepressants, antioxidants, anxiolytics, cytotoxic, sedatives, thrombolytics

## Abstract

The research focused on *Wedelia montana* (Blume) Boerl because of its numerous medicinal applications. *W. montana* belongs to the Asteraceae family. This investigation is intended to analyze the phytochemical content of the methanol extract of *W. montana* (MEWM) and evaluate its biological features by utilizing in vitro and in vivo models. In vitro examinations determined antioxidant capability, cytotoxic, anthelmintic, and thrombolytic activity of the MEWM. Furthermore, in vivo research included testing for the effects of antidepressants (TST and FST), effects on anxiety (LDT, HBT, and EPM), activities of sedatives (HCT and OFT), and analgesic activities (formalin‐induced licking test and tail immersion test). Treatment with MEWM exhibited potent antioxidant properties, with a cytotoxicity test revealing an LC_50_ value of 256.2 μg/mL, in contrast to 142.28 μg/mL for the positive control. It also resulted in the shortest times for paralysis and mortality at the highest dosage in the anthelmintic assay and notable thrombolytic activity (*p* < 0.0001). Moreover, MEWM has shown considerable efficacy contingent upon the FST, TST, EPM, HBT, and LDT dose and sedative effects in the OFT and HCT. A 200 mg/mL dosage in the analgesic assessment had no significant impact on the tail immersion test. However, MEWM demonstrated substantial analgesic action in the formalin‐induced paw‐licking experiment (*p* < 0.0001). The data indicate that MEWM is a potential source of antioxidant, cytotoxic, anthelmintic, thrombolytic, antidepressant, anxiolytic, sedative, and antinociceptive compounds. Further research is necessary to comprehend its therapeutic benefits completely.

## 1. Introduction

Natural products with varied bioactive components and comparatively low toxicity have drawn more interest from all around the world [1]. Historically, people across cultures have relied on plant‐based remedies to treat a wide array of illnesses [2]. According to the World Health Organization (WHO), over 80% of the global population still relies on herbal or plant‐derived medications as a critical component of healthcare [3].

In connection with this, numerous diseases, including cancer, atherosclerosis, cardiovascular disease, diabetes, and metabolic disorders, are brought on by oxidative stress [4]. Our bodies naturally fight against free radical damage using protective compounds such as vitamin C (ascorbic acid), vitamin E (tocopherol), and glutathione [5]. However, when these defense systems are compromised, external supplementation with antioxidants becomes necessary to protect cellular health.

Since cancer is still one of the world’s top causes of death, considerable efforts have been made to create effective ways to fight this illness. A key component of contemporary cancer treatment is chemotherapy, which is used to treat solid tumors, lymphoma, and leukemia with a variety of therapeutically available medications [6]. Alongside anticancer research, plants have also been traditionally used for their anthelmintic properties. Secondary metabolites such as terpenes, glycosides, flavonoids, saponins, tannins, and alkaloids contribute to the expulsion or destruction of parasitic worms and support gastrointestinal health [7]. Moreover, thrombosis is a blood clot that can result in coronary blood irregularities, including deadly brain hemorrhages and acute myocardial infarction. The first‐line treatment for thrombus is intravenous heparin because of its effectiveness, safety, and viability. Streptokinase and urokinase are commonly used because they are less costly than other thrombolytic medications, but they are also risky because they can result in severe bleeding, reocclusion, and reinfarction [8].

Turning to mental health, there is growing concern about the rising incidence of depression, anxiety, and sleep disorders [9]. While traditionally linked to low levels of brain chemicals like serotonin, recent research suggests that oxidative stress and elevated reactive oxygen species (ROS) may also contribute significantly to depression [10, 11]. Despite being widely available, current antidepressants often have limited effectiveness and significant side effects, highlighting the need for better treatments [12]. Anxiety is the most common psychiatric disorder, affecting 10%–25% of people, and sleep disturbances, affecting over 30%, are the second most frequent sign of psychological distress [13]. According to the WHO, benzodiazepines like diazepam are essential for treating sleep and anxiety disorders due to their effectiveness and safety. However, diazepam can cause serious side effects such as aggression, disinhibition, hypotension, and respiratory issues, particularly at high doses. This underscores the need for new treatments that are safer and more effective. In addition to neurological treatments, analgesics are another important class of drugs used to manage pain without causing mental confusion or loss of consciousness, unlike anesthetics [14]. These drugs act on either the central or peripheral nervous systems, offering relief from pain while preserving neurological function [15].

*Wedelia montana* (Blume) Boerl. belongs to the Asteraceae family. It is widely distributed in the shaded, moist areas of Nepal, India, China, and the nation of Bangladesh. In Bangladesh, *W. montana* has acclimatized to occupy scanty woodlands, wet meadows, streams, and seasonally inundated lowland forests, including Chittagong, Cox’s Bazar, Moulvibazar, and Sylhet. In the Caribbean and Central America, traditional medicine uses the genus *Wedelia*, namely, *Wedelia trilobata*, to treat back discomfort, cramping in the muscles, rheumatism, obstinate wounds, sores, swelling, and arthritic and aching joints. It has been found that the aerial portions exhibit antibacterial action against *Pseudomonas aeruginosa* and *Bacillus subtilis* [16]. Despite its traditional use and widespread application in folk medicine, *W. montana* lacks scientific validation, with no published data to date on its pharmacological activities. To address this gap, the present study aims to systematically investigate its potential therapeutic properties through a combination of in vitro and in vivo experimental models, thereby providing a scientific basis for its ethnomedicinal use and identifying promising leads for future drug development.

## 2. Materials and Methods

### 2.1. Chemicals and Reagents

All chemicals and reagents used in this study were of high analytical grade and obtained from reputable commercial suppliers to ensure experimental accuracy and consistency. Key solvents and reagents were sourced from companies such as Merck (India), Sigma Chemical Co. (USA), Fluka Chemical GmbH (Switzerland), BDH Chemical Ltd. (UK), and Riedel‐De Haen AG (Germany). Pharmaceutical standards, including streptokinase, diclofenac sodium, tramadol, vincristine sulfate, fluoxetine hydrochloride, and diazepam, were collected from authorized Bangladeshi pharmaceutical companies. All materials were handled following standard laboratory protocols to ensure the integrity and reliability of the study.

### 2.2. Plant Collection, Identification, Verification, and Extract Preparation

On January 16, 2024, fresh *W. montana* leaves were collected from the Bishocolony Hill area in Chattogram, Bangladesh (22.375519, 91.785425) at 27°C. The plant’s identity was verified by Dr. Shaikh Bokhtear Uddin, a taxonomist at the University of Chittagong, where a voucher specimen (accession number: MHM 25052407) is preserved at the university herbarium (CTGUH). The leaves were shade‐dried and ground to a fine powder (1‐mm mesh), and 400 g of the powder was extracted in methanol (HPLC grade) at a 6:1 ratio (solvent to plant material) for 72 h at 26 ± 1°C [17, 18]. The extract was filtered using a Whatman No. 1 filter paper and concentrated under reduced pressure using a rotary evaporator. A total of 26 g of crude methanol extract was obtained and stored at 4°C for subsequent analysis.

### 2.3. Experimental Animals and Ethical Statements

*Swiss albino* mice, aged 5–6 weeks and weighing between 21 and 30 g, were sourced from the International Centre for Diarrheal Disease and Research (ICDDR, B), Bangladesh. The mice were housed under controlled laboratory conditions, with access to fresh water and standard food, a 12‐h light/dark cycle, and maintained at 25 ± 1°C and 60 ± 5% humidity. Before the experiment, the mice were acclimated for 48 h [19]. All procedures followed the 1995 Principles for Research Studies on Animals and Moral Guidelines by the Swiss Academy of Medical Sciences and the Swiss Academy of Sciences [20]. The study was reviewed and approved by the Planning and Development (P&D) Committee (Pharm‐P&D‐61/08‐16–122) of the Pharmacy Department at the International Islamic University Chittagong, Bangladesh.

### 2.4. Acute Toxicity Study

The acute toxicity study followed OECD guidelines 423, using seven groups of *Swiss albino* mice (*n* = 6 per group). The control group was given 1% Tween 80 in distilled water, while the experimental groups received oral methanol extract of *W. montana* (MEWM) at doses of 200 to 4000 mg/kg. Mice were provided with standard food and water and monitored for behavioral changes, allergic reactions, and mortality over 72 h and 14 days [21, 22].

### 2.5. Phytochemical Screening

MEWM underwent qualitative phytochemical analysis utilizing a previously established methodology [22] to identify secondary metabolites such as flavonoids, phenolic compounds, tannins, phlobatannins, saponins, and carboxylic acids.

### 2.6. Antioxidant Activity

#### 2.6.1. DPPH Radical Scavenging Activity

The study assessed the antioxidant activity of MEWM using the DPPH free radical scavenging assay based on the Brand‐Williams method [23]. Absorbance measurements were conducted using a Shimadzu Biospec 1601 UV–visible spectrophotometer. Various concentrations of MEWM (ranging from 31.25 to 500 μg/mL) were mixed with a DPPH solution and compared to a reference antioxidant, ascorbic acid. After 30 min of incubation at room temperature, absorbance was measured at 517 nm. The reduction in absorbance indicated the antioxidant (radical scavenging) potential of MEWM, expressed as a percentage of inhibition:

(1) % scavenging  activity=Ac−AsAc×100,

where *A* *c* is the absorbance of the control and *A* *s* is the absorbance of the test sample (MEWM or ascorbic acid).

#### 2.6.2. Reducing Power Capacity

The reducing power of the *W. montana* methanol extract was evaluated using a modified Oyaizu method [24]. Extract concentrations ranging from 31.25 to 500 μg/mL were mixed with phosphate buffer (pH 6.6) and potassium ferricyanide and then incubated at 50°C for 20 min. After adding trichloroacetic acid, the mixtures were centrifuged, and the supernatant was combined with distilled water and ferric chloride. Absorbance was measured at 700 nm (Shimadzu Biospec 1601 UV–visible spectrophotometer), with higher absorbance indicating stronger reducing power. Ascorbic acid served as the standard, and phosphate buffer was used as the blank.

### 2.7. Total Phenolic Content

The total phenolic content of the extract was determined using the Folin–Ciocalteu method, with gallic acid as the standard. A calibration curve was prepared using gallic acid solutions (100–800 μg/mL), mixed with diluted Folin–Ciocalteu reagent and sodium carbonate. After 30 min of incubation at 25°C, absorbance was measured at 765 nm (Shimadzu Biospec 1601 UV–visible spectrophotometer). The extract was tested similarly, and phenolic content was expressed as milligrams of gallic acid equivalents (mg GAE) per gram of extract [25].

### 2.8. Total Flavonol Content

The total flavonol content was measured using a colorimetric method involving AlCl_3_ and sodium acetate, with absorbance recorded at 440 nm after 2 h and 15 min at 20°C. Results were calculated using a quercetin calibration curve and expressed as milligrams of quercetin equivalents (mg QE) per gram of dry extract [26].

### 2.9. Total Flavonoid Content

The total flavonoid content of the MEWM was measured utilizing the specified technique [26]. A mixture of 3 mL methanol and 1 mL of *W. montana* extract or standard was prepared at varying concentrations. Then, 200 μL of 10% aluminum chloride and 200 μL of 1M potassium acetate were added. After incubating for 30 min at room temperature, the absorbance was measured at 420 nm using a UV spectrophotometer (Shimadzu Biospec 1601), with methanol as the control and a blank as reference. The flavonoid content in the extracts was quantified in mg/g QE.

### 2.10. In Vitro Brine Shrimp Cytotoxicity

A lethality experiment using brine shrimp was conducted to evaluate the cytotoxic potential of various plant extracts [27]. To simulate saltwater, 38 g of NaCl was dissolved in 1000 mL of distilled water, with NaOH added to maintain a consistent pH. Brine shrimp eggs were hatched in this synthetic saltwater to produce nauplii. Dimethyl sulfoxide (DMSO) was used to prepare test samples at varying concentrations (25–100 μg/mL). Colchicine served as the positive control at the same concentration range, while DMSO acted as the negative control. Nauplii were visually counted at room temperature (25°C) and transferred into vials containing 5 mL of simulated saltwater. Test samples at different concentrations were added using a micropipette. After 24 h, the surviving nauplii were counted to assess the cytotoxic effects of the extracts. This method provided a simple and effective way to screen for cytotoxic activity.

(2) % mortality=number of nauplii taken−number of nauplii aliveNumber of nauplii taken×100.

### 2.11. In Vitro Anthelmintic Activity

The anthelmintic efficacy of the crude MEWM was evaluated using a modified technique [28]. *Tubifex tubifex* worms (2–2.5 cm), resembling human intestinal roundworms, were divided into three groups: a distilled water control, levamisole (0.5–1 mg/mL), and plant extract (5–10 mg/mL). Each Petri dish contained 10–12 worms with 3 mL of test solution. Paralysis and death times were recorded, with paralysis defined as no movement except shaking, and death confirmed by lack of response to agitation or warm water.

### 2.12. In Vitro Thrombolytic Activity

The method of Prasad et al. [29], with slight modifications, was employed to assess the in vitro clot lysis potential of the extract. In this thrombolytic assay, 7 mL of venous blood was collected from five healthy, nonsmoking volunteers with no history of anticoagulant use. Initially, 1 mL of blood was transferred into preweighed sterile tubes and incubated at 37°C for 45 min to facilitate clot formation. After incubation, the weight of the clots was measured. To evaluate clot lysis, each tube received 100 μL of either the test extract (1 mg/mL), streptokinase (positive control), or distilled water (negative control). The tubes were then reincubated at 37°C for 90 min to allow clot dissolution. The extent of thrombolysis was determined by measuring the change in clot weight, with a reduction in weight indicating clot lysis. The percentage of clot lysis was calculated using the weight difference and expressed as follows [30]:

(3) %  of  clot  lysis=weight  of   released  clotclot  weight×100.

### 2.13. Antidepressant Activity

#### 2.13.1. Forced Swimming Test (FST)

This method was used to assess the antidepressant effects of MEWM in *Swiss albino* mice [31, 32]. Mice were individually placed in transparent cylinders (10 cm height × 25 cm width) filled with 19 cm of water at 25 ± 1°C. Behavior was recorded for 6 min, with immobility measured during the last 4 min.

#### 2.13.2. Tail Suspension Test (TST)

The TST is considered a reliable method for evaluating antidepressant activity [31, 33]. Mice were suspended 50 cm above the ground by the tail using adhesive tape, positioned about 1 cm from the tip. The test was recorded for 6 min, with immobility measured during the last 4 min, following a 2‐min adjustment period.

### 2.14. Anxiolytic Activity

#### 2.14.1. Elevated Plus Maze (EPM) Test

The EPM is a critical tool for researching the neuroprotective and anxiolytic properties of test drugs. The EPM, elevated 40 cm above the ground, has a plus‐shaped structure with two open arms (25 × 5 cm^2^) aligned perpendicularly and two closed arms (25 × 5 cm^2^ with 16‐cm walls). Each test subject was placed in the center of the maze, facing an open arm, and a stopwatch was started. Over 5 min, several parameters were observed: the initial preference of mice for closed or open arms, the number of entries into both types of arms, and the time spent in each. An entry was defined as all four paws of the mouse being inside an arm. After the initial observation, animals were administered different treatments: diazepam for the standard group, saline for the control group, and two test samples of MEWM. A 30‐min break was given posttreatment before retesting in the maze. During the subsequent 5‐min observation, the time spent in open and closed arms was recorded to assess changes in behavior. This process allowed the evaluation of the anxiolytic effects of the test drugs [34].

#### 2.14.2. Hole Board Test (HBT)

The test apparatus consisted of a wooden box (20 × 40 cm) with 16 pegs, each raised 15 cm from the floor. The centers of the two holes were spaced 10 cm apart, and the base was 25 cm above the ground [19, 35]. After a 30‐min treatment period, mice were placed in the central enclosure to move freely. Head dips through the holes were recorded for 5 min, with the first minute for adjustment.

#### 2.14.3. Light–Dark Test (LDT)

The light–dark box test helps assess anxiety or anxiogenic behavior in mice. It consists of a square container (46 × 27 × 30 cm) divided into a small (18 × 27 cm) and a larger area (27 × 27 cm), with a 7.5 × 7.5 cm opening between them [36]. The white compartment was lit by an 80‐W lamp, and the time spent in each section was recorded over 5 min, with 1 min for adjustment. The environment was dim during the test [37].

### 2.15. Sedative Activity

#### 2.15.1. Open Field Test (OFT)

Treatments were administered following Section 2.3 after the mice were divided. After receiving their different treatments, each mouse was put on a board and observed for three minutes, during which time the number of squares it traversed was recorded. Additionally, at 30, 60, 90, and 120 min after treatment, the mobility across squares was monitored [38].

#### 2.15.2. Hole Cross Test (HCT)

Stainless steel was used to make the hole cross cage, which measures 30 cm × 20 cm × 14 cm. A divider with a 3‐cm gap and a 7.5‐cm height was positioned in the middle of the cage. The mice positioned themselves in the center of each side of the cross device with holes. A video camera was used to record the number of holes they passed through between chambers, and the results were then assessed every three minutes between 0 and 120 min [39].

### 2.16. Antinociceptive Activity

#### 2.16.1. Tail Immersion Test (TIT)

A TIT was used to investigate the analgesic mechanism [40]. The last 1–2 cm of the tail of *Swiss albino* mice was immersed in hot water to induce pain responses. Mice that retracted their tails within 5 s during a sensitivity test were selected. After a 16‐h fast with water access, animals were treated with tramadol as the standard. Latency, the time to retract the tail from hot water (55 ± 1°C), was measured for up to 120 min, with 15 s as the cutoff to prevent injury.

#### 2.16.2. Formalin‐Induced Pain Test

A biphasic approach has been used in the murine model and examined as indicated in [41]. Mice received a 20‐μL formalin injection (2.5%) to the subplantar area of their hind paw, following intraperitoneal pretreatment with saline (0.1 mL/kg), diclofenac sodium (10 mg/kg), or the extract (200 and 400 mg/kg). The first phase (0–5 min) reflected neurogenic pain, while the second phase (15–30 min) indicated inflammatory pain. Antinociceptive activity was measured by the length of time spent licking. Treatments followed the protocol in Section 2.3.

### 2.17. Statistical Analysis

The results are presented as mean ± SEM (*n* = 5 for in vivo experiments; *n* = 3 for in vitro anthelmintic assays). Statistical analysis was carried out using SPSS v16.0 (IBM Corp., USA) [42], with one‐way ANOVA followed by Dunnett’s post hoc test for intergroup comparisons. Significance levels were set at ∗*p* < 0.05, ^∗∗^ *p* < 0.01, ^∗∗∗^ *p* < 0.001, and ^∗∗∗∗^ *p* < 0.001, with all treated groups compared against the control or standard reference group. All graphical representations were prepared using GraphPad Prism 8.0.1 to ensure clarity and consistency in data visualization.

## 3. Results

### 3.1. Qualitative Phytochemical Analysis

MEWM qualitative phytochemical profile showed the presence of alkaloids, phenolic, reducing sugars, cardiac glycosides, flavonoids, quinones, carboxylic acid, and resins (Table 1).

**Table TABLE 1:** Qualitative phytoconstituents screening of MEWM.

Phytochemical class	Test performed	Results
Alkaloids	Mayer’s test	+
	Wagner’s test	+

Carbohydrates	Barfoed’s test	−

Reducing sugars	Benedict’s test	+
	Fehling’s test	+

Glycosides	10% NaOH test	−

Cardiac glycosides	Keller–Killani test	+

Proteins and amino acids	Xanthoproteic test	−

Flavonoids	Alkaline reagent test	+
	Lead acetate test	+

Phenolic compounds	Iodine test	+

Tannins	Braymer’s test	−
	10% NaOH test	−

Phlobatannins	HCl test	−

Saponins	NaHCO_3_ test	−

Phytosterols	Acetic anhydride test	−

Triterpenoids	Salkowski’s test	−

Quinones	Conc. HCl test	+

Anthocyanins	HCl test	−

Carboxylic acid	NaOH test	+

Resins	Acetic anhydride test	+

*Note:* The symbol (+) indicates the presence, and the symbol (−) indicates the absence of phytochemical groups.

### 3.2. Acute Toxicity Test

Acute toxicity testing showed that oral administration of MEWM at doses ranging from 200 to 4000 mg/kg in mice was safe. No toxic symptoms were observed within 72 h or during the 14‐day monitoring period. As a result, 200 and 400 mg/kg doses were selected for the current study.

### 3.3. In Vitro Antioxidant Activity

#### 3.3.1. DPPH Radical Scavenging Activity

The MEWM exhibited (Figure 1) high to moderate antioxidant activity in DPPH free radical scavenging, with activity increasing with dose. The IC_50_ values for MEWM and ascorbic acid were 211.23 and 198.75 μg/mL, respectively.

**Figure FIGURE 1:**
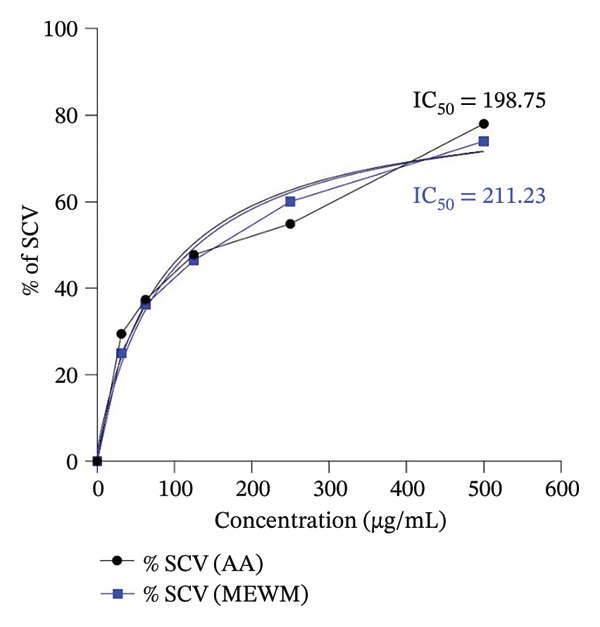
Calibration curve of ascorbic acid and MEWM for the DPPH free radical scavenging. MEWM = methanol extract of *Wedelia montana*; IC_50_ = 50% inhibitory concentration; AA = ascorbic acid.

#### 3.3.2. Reducing Capacity

The results indicated a proportionate enhancement in reducing power corresponding to the rise in the extract content (Figure 2).

**Figure FIGURE 2:**
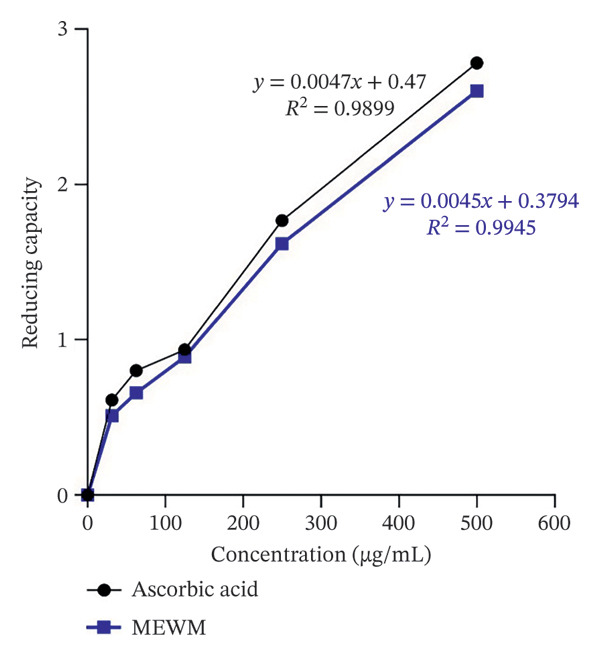
Methanol extract of *Wedelia montana* reducing capacity, the capacity reduction with concentration.

### 3.4. Total Phenolic Content

The crude extract was evaluated for its total phenolic content. Table 2 presents the phenolic potentials of MEWM, recorded as 154.483 ± 2.118 GAE mg/g.

**Table TABLE 2:** Determination of total phenolic content.

Sl. no.	Concentration (μg/mL)	Weight of dry extract per mL (gm)	Absorbance at 765 nm	GAE conc. C (μg/mL)	GAE conc. C (mg/mL)	TPC as GAE *A* = (*c* × *v*)/*m* (mg/gm)	Mean ± SEM (mg/gm)
1	1000	0.001	0.907	154.26	0.154	154.26	154.48 ± 2.11
2	1000	0.001	0.913	158.26	0.158	158.26	
3	1000	0.001	0.902	150.93	0.150	150.93	

*Note:* Values are presented as mean ± SEM (*n* = 3).

### 3.5. Total Flavonol Content

The extract’s total flavonol concentration was quantified as 409.5 ± 5.81 QE mg/g of dry extract, as shown in Table 3.

**Table TABLE 3:** Determination of total flavonol content.

Sl no.	Concentration (μg/mL)	Weight of dry extract per mL (gm)	Absorbance at 440 nm	QE conc. C (μg/mL)	QE conc. C (mg/mL)	TFC as QE *A* = (*c* × *v*)/*m* (mg/gm)	Mean ± SEM (mg/gm)
1	1000	0.001	0.51	419.2	0.41	419.2	409.5 ± 5.81
2	1000	0.001	0.50	410.2	0.41	410.2	
3	1000	0.001	0.49	399.1	0.39	399.1	

*Note:* Values are shown as mean ± SEM (*n* = 3).

### 3.6. Total Flavonoid Content

The total flavonoid content of the extract was quantified using the QE calibration curve, yielding a value of 295.22 ± 5.614 mg/g of dry extract, as shown in Table 4.

**Table TABLE 4:** Determination of total flavonoid content.

Sl no.	Concentration (μg/mL)	Weight of dry extract per mL (gm)	Absorbance at 415 nm	QE conc. C (μg/mL)	QE conc. C (mg/mL)	TPC as QE *A* = (*c* × *v*)/*m* (mg/g)	Mean ± SEM (mg/gm)
1	1000	0.001	0.811	292.55	0.292	292.55	295.22 ± 5.61
2	1000	0.001	0.823	306	0.306	306	
3	1000	0.001	0.806	287.11	0.287	287.11	

*Note:* Values are shown as mean ± SEM (*n* = 3).

### 3.7. In Vitro Brine Shrimp Cytotoxicity

The brine shrimp lethality assay showed (Figure 3) MEWM’s LC_50_ at 256.2 μg/mL, indicating lower cytotoxicity compared to the positive control, vincristine sulfate, with an LC_50_ value of 142.28 μg/mL.

**Figure FIGURE 3:**
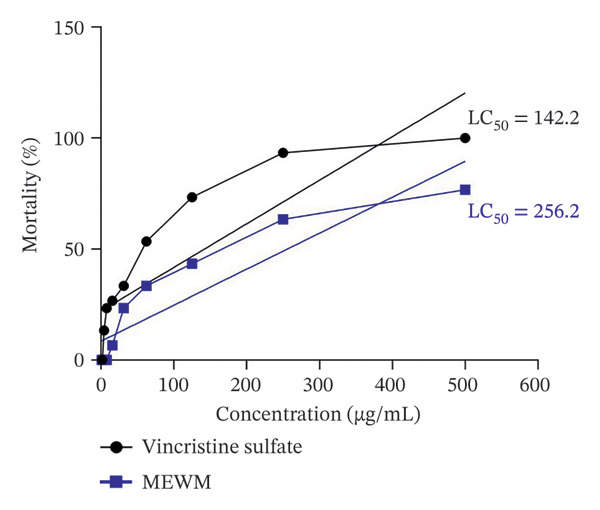
Cytotoxic effects of MEWM. The comparative mortality rates of different concentrations of MEWM and the reference cytotoxic agent, vincristine sulfate, were analyzed using regression analysis.

### 3.8. In Vitro Anthelmintic Activity

MEWM showed significant anthelmintic activity (Figure 4) against *T. tubifex* worms, with increasing paralysis and mortality times at higher concentrations. Levamisole, a positive control, exhibited stronger effects at lower concentrations.

**Figure FIGURE 4:**
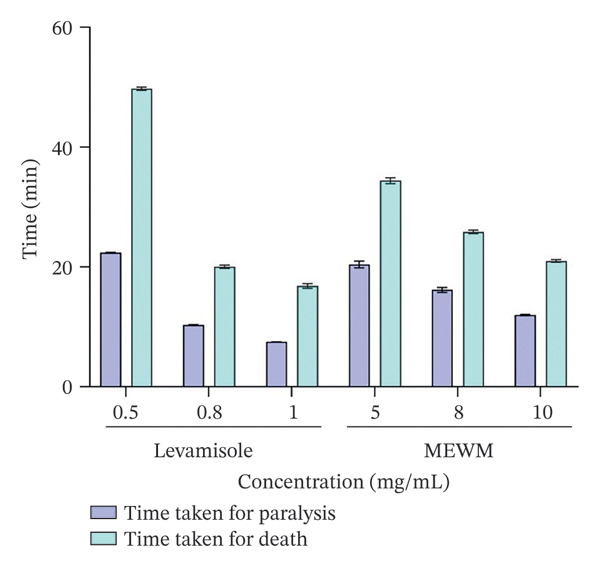
Anthelmintic activity of the methanol extract of *Wedelia montana*. Values are shown as mean ± SEM (*n* = 3).

### 3.9. In Vitro Thrombolytic Activity

MEWM and streptokinase demonstrated thrombolytic activity of 35.06 ± 0.20% and 73.39 ± 0.98%, significantly (*p* < 0.0001) higher than the negative control (distilled water, 6.66 ± 0.71%), presented in Figure 5.

**Figure FIGURE 5:**
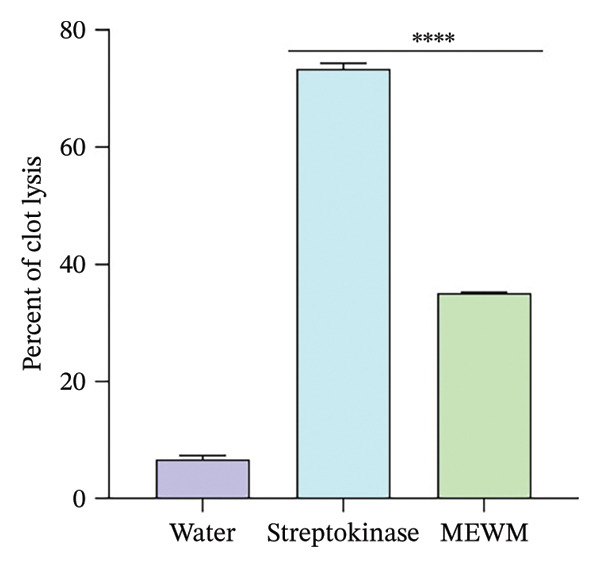
In vitro clot lysis by water, MEWM, and streptokinase. The Dunnett test follows ANOVA, and values are reported as mean ± SEM with ^∗∗∗∗^ *p* < 0.0001, a significant difference from the control.

### 3.10. Antidepressant Activity

As shown in Figures 6 and 7, MEWM doses of 200 and 400 mg/kg significantly reduced immobility times (*p* < 0.0001) in both FST and TST.

**Figure FIGURE 6:**
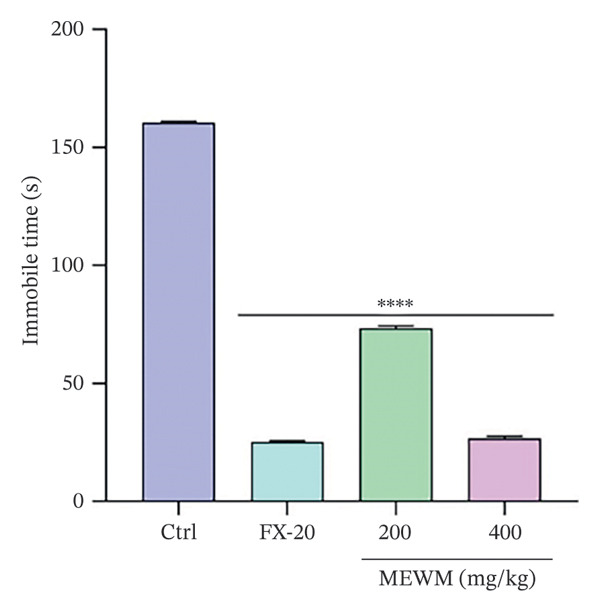
The impact of MEWM on the mice forced swimming test immobility time. The findings were presented as mean ± SEM ^∗∗∗∗^ *p* < 0.0001, indicating statistical significance compared to the control group.

**Figure FIGURE 7:**
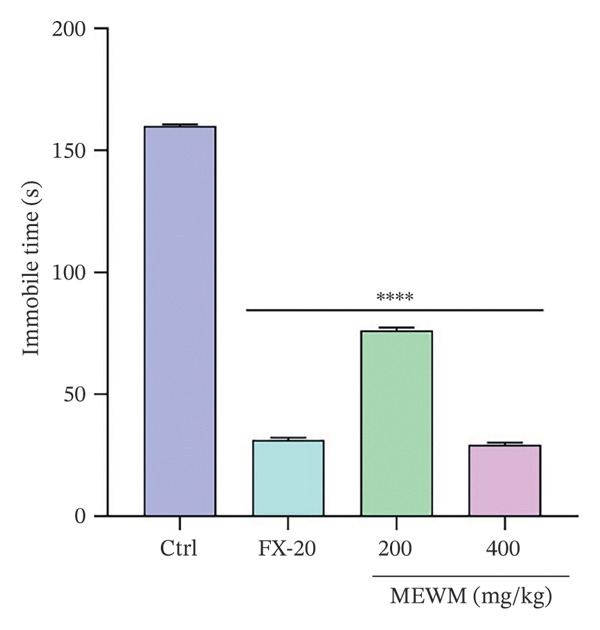
The effects of MEWM treatment on the duration of immobility time for TST. The findings were presented as mean ± SEM ^∗∗∗∗^ *p* < 0.0001, indicating statistical significance compared to the control group.

### 3.11. Anxiolytic Activity

MEWM at 200 and 400 mg/kg significantly increased open arm duration in the EPM test (Figure 8) and head dipping in the HBT (Figure 9), similar to diazepam. In the light–dark box test, MEWM prolonged time in the light chamber and reduced time in the dark chamber, showing significant effects (*p* < 0.0001) compared to the control group and similar results to the reference medication.

**Figure FIGURE 8:**
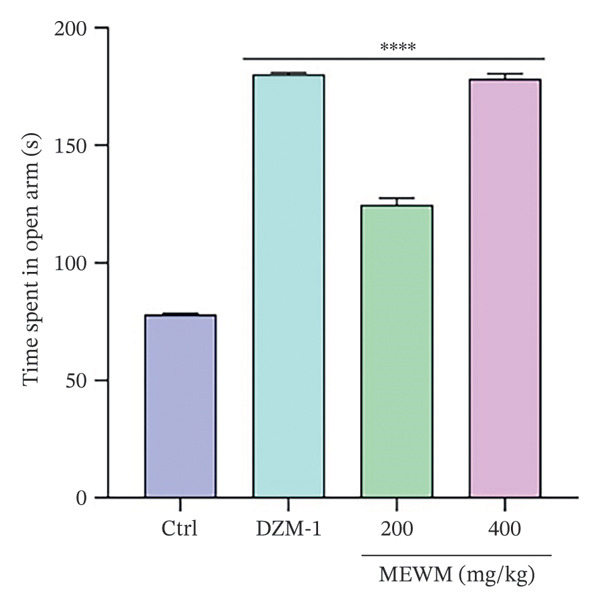
MEWM affects how long an arm is left open during an EPM test. The data were presented as mean ± SEM with statistical significance determined by comparing the groups (^∗∗∗∗^ *p* < 0.0001).

**Figure FIGURE 9:**
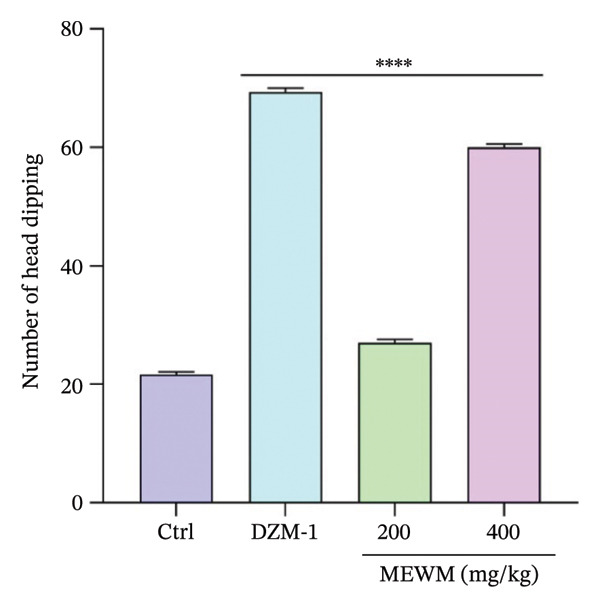
MEWM effects on the hole board test. The data were presented as mean ± SEM, with statistical significance determined by comparing the groups (^∗∗∗∗^ *p* < 0.0001).

### 3.12. Sedative Activity

In both the OFT and HCT, MEWM significantly reduced the locomotor activity of mice at both 200 and 400 mg/kg doses, similar to the effects of diazepam (DZM‐1). In the OFT, block crossings decreased markedly (*p* < 0.0001), indicating reduced general mobility (Table 5). Likewise, in the HCT, hole crossings significantly declined across all observation periods (30–120 min) with strong statistical significance (*p* < 0.0001, *p* < 0.001, and *p* < 0.01), suggesting dose‐dependent suppression of spontaneous movement by MEWM (Table 6).

**Table TABLE 5:** Sedative effect of MEWM in the open field test.

Groups	Dose	The number of squares traveled				
		0 min	30 min	60 min	90 min	120 min
Control	10 mL/kg	80 ± 0.57	67 ± 0.5	48 ± 0.57	53.5 ± 0.76	59.83 ± 0.74
DZM‐1	1 mg/kg	70.83 ± 0.60	56.16 ± 0.60^∗∗∗∗^	26.83 ± 0.60^∗∗∗∗^	17 ± 0.57^∗∗∗∗^	14 ± 0.57^∗∗∗∗^
MEWM	200 mg/kg	92.5 ± 0.76	81.83 ± 1.07^∗∗∗∗^	61.66 ± 0.88^∗∗∗∗^	66.66 ± 0.84^∗∗∗∗^	74.5 ± 0.76^∗∗∗∗^
MEWM	400 mg/kg	58.66 ± 0.88	41.66 ± 0.71^∗∗∗∗^	21.5 ± 0.76^∗∗∗∗^	18 ± 0.96^∗∗∗∗^	11.66 ± 0.76^∗∗∗∗^

*Note:* Values are expressed as mean ± SEM, and statistical significance was defined as ^∗∗∗∗^ *p* < 0.0001 when compared to the control group.

**Table TABLE 6:** Sedative effect of MEWM in the hole cross test.

Groups	Dose (mg/kg)	The number of holes crossed				
		0 min	30 min	60 min	90 min	120 min
Control	10 mL/kg	18 ± 0.57	18.83 ± 0.60	23 ± 0.57	21 ± 0.57	18 ± 0.57
DZM‐1	1	8.16 ± 0.60	7 ± 0.57^∗∗∗∗^	6 ± 0.57^∗∗∗∗^	5 ± 0.57^∗∗∗∗^	3 ± 0.57^∗∗∗∗^
MEWM	200	21.33 ± 0.76	22 ± 0.68^∗∗^	26.66 ± 0.66^∗∗∗^	24.66 ± 0.66^∗∗∗^	21.83 ± 0.60^∗∗∗^
MEWM	400	6 ± 0.57	5.16 ± 0.60^∗∗∗∗^	4 ± 0.57^∗∗∗∗^	5.33 ± 0.33^∗∗∗∗^	3 ± 0.57^∗∗∗∗^

*Note:* Values are expressed as mean ± SEM, and statistical significance was defined as ^∗∗^ *p* < 0.0046, ^∗∗∗^ *p* < 0.0010, and ^∗∗∗∗^ *p* < 0.0001 when compared to the control group.

### 3.13. Antinociceptive Activity

#### 3.13.1. TIT

The latency time from the initial measuring phase (0 min) to the concluding measuring phase (120 min) in the TIT reflected the activity of the experimental mice. In comparison to the control group, the total mobility of the experimental mice was significantly reduced (*p* < 0.0001) at tramadol at the tested dosages (10 and 400 mg/kg). In contrast, the tested MEWM 200 mg/kg dose showed significance only at 30 min (*p* < 0.0001) (Table 7).

**Table TABLE 7:** Antinociceptive effect of MEWM in the tail immersion test.

Groups	Dose (mg/kg)	Latency time (s)				
		0 min	30 min	60 min	90 min	120 min
Control	10 mL/kg	1.28 ± 0.007	1.35 ± 0.013	1.28 ± 0.002	1.31 ± 0.012	1.33 ± 0.014
Tramadol	10	1.33 ± 0.015	4.58 ± 0.011^∗∗∗∗^	5.88 ± 0.017^∗∗∗∗^	5.04 ± 0.109^∗∗∗∗^	4.44 ± 0.084^∗∗∗∗^
MEWM	200	1.39 ± 0.018	1.46 ± 0.018^∗∗∗^	1.41 ± 0.015	1.44 ± 0.012	1.48 ± 0.006
MEWM	400	1.40 ± 0.013	3.51 ± 0.019^∗∗∗∗^	4.33 ± 0.182^∗∗∗∗^	3.62 ± 0.018^∗∗∗∗^	3.09 ± 0.055^∗∗∗∗^

*Note:* The mean ± SEM was used to express the results, and statistical significance was defined as ^∗∗∗^ *p* < 0.0010, and ^∗∗∗∗^ *p* < 0.0001 when compared to the control group.

#### 3.13.2. Formalin‐Induced Paw Licking

Figure 10 illustrates the outcomes of the formalin‐induced paw‐licking assay in mice. Mice treated with diclofenac sodium (10 mg/kg) and MEWM (200, 400 mg/kg) significantly (*p* < 0.0001) reduced paw‐licking duration during both acute and delayed phases in comparison with the control group.

**Figure FIGURE 10:**
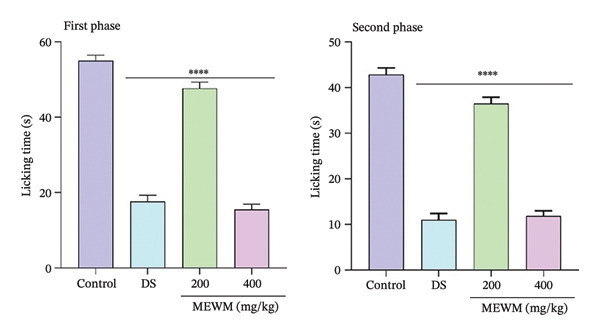
First and second phase formalin test results for MEWM and DS. The values significantly differ from the control group with ^∗∗∗∗^ *p* < 0.001, and the data were presented as mean ± SEM.

## 4. Discussion

Since ancient times, natural remedies have attracted interest for their healing properties. Traditional herbal medicines—sourced from plants, minerals, and organic compounds—remain vital in developing treatments, supplements, and products across healthcare, nutrition, and wellness sectors [43]. In recent years, ethnomedicine—the study of traditional knowledge and medicinal plant use—has gained renewed attention as a source of novel bioactive compounds. This study investigates the ethnomedicinal uses of *W. montana*, highlighting its bioactive constituents and potential as a natural treatment for various ailments.

The remarkable pharmacological effects of medicinal plants stem from their diverse secondary metabolites. This study’s qualitative phytochemical analysis of *W. montana* revealed key constituents such as alkaloids, phenolics, reducing sugars, cardiac glycosides, flavonoids, quinones, carboxylic acids, and resins. These compounds show promise in various therapeutic areas, including anthelmintic, neuropharmacological, cytotoxic, antioxidant, and antinociceptive applications, underscoring *W. montana*’s potential and versatility as a natural medicine.

The antioxidant activity of MEWM was demonstrated through DPPH scavenging, where the extract showed a notable IC_50_ value. Flavonoids and phenolic compounds can chelate metal ions, inhibit lipid peroxidation, and modulate antioxidant enzymes, collectively reducing oxidative stress at the cellular level [44]. High levels of total phenolics and flavonoids support this result, suggesting strong radical scavenging and hydrogen‐donating abilities. These findings indicate the extract’s potential in mitigating oxidative stress, which plays a role in various chronic diseases.

The brine shrimp lethality assay findings indicate low toxicity, supporting its potential safety for pharmaceutical use. While values below 250 μg/mL are typically considered cytotoxic, this near‐threshold value still suggests the need for further evaluation. The cytotoxic properties of MEWM may be attributed to the presence of alkaloids, flavonoids, and phenolic compounds. Alkaloids and phenolics, in particular, are known to induce apoptosis and disrupt cell cycle progression, indicating potential applications in anticancer therapy [45].

In anthelmintic assays, MEWM exhibited dose‐dependent effects, ranging from paralysis to death in helminths, albeit at higher doses than levamisole, likely due to its complex phytochemical composition. Flavonoids, condensed tannins, and polyphenolic compounds are key contributors to its activity, aligning with previous findings on their role in helminth inhibition [42].

Thrombolytic agents impede thrombus formation using plasmin, a naturally occurring fibrinolytic substance that dissolves clots via the hydrolysis of fibrinogen and fibrin [46]. The thrombolytic potential of MEWM appears to be driven by its capacity to modulate the fibrinolytic system. Flavonoids and alkaloids may enhance the activity of plasmin (primary clot‐dissolving enzyme) by stimulating plasminogen conversion or inhibiting fibrinolysis inhibitors such as PAI‐1. These compounds may also reduce vascular inflammation and platelet aggregation, supporting MEWM’s potential in managing thrombotic disorders.

Behavioral studies (FST, TST, EPM, HBT, and LDT) showed that MEWM has antidepressant and anxiolytic effects. It significantly reduced immobility in FST and TST, comparable to fluoxetine, suggesting serotonergic involvement. These effects are likely due to alkaloids and flavonoids, which modulate neurotransmitters, inhibit MAO, and boost serotonin, dopamine, and norepinephrine levels [47, 48]. The anxiolytic effects of MEWM were evidenced by increased open‐arm time (EPM), head‐dipping (hole board), and compartment transitions (LDT), indicating reduced anxiety. These effects likely involve GABAergic and glutamatergic modulation, possibly via benzodiazepine‐like action on GABA‐A receptors. Flavonoids, alkaloids, and phenols likely work synergistically to reduce neuronal excitability and stress responses, contributing to CNS depressant activity [49, 50]. Moreover, the sedative effects of MEWM, demonstrated through reduced locomotor activity, may also involve GABAergic pathways. Flavonoids can act as positive allosteric modulators at GABA‐A receptor sites, enhancing chloride ion influx into neurons and thereby producing CNS depressant effects. This mechanism parallels that of diazepam and underscores the calming influence of MEWM on the central nervous system [39].

In analgesic assessments, MEWM significantly prolonged reaction times in the TIT, indicating peripheral analgesic action. The formalin test further confirmed both central and peripheral analgesic effects, as MEWM reduced licking behavior during early (neurogenic) and late (inflammatory) phases. These biphasic effects resemble the actions of opioids and NSAIDs, suggesting a broad analgesic potential [51, 52]. MEWM likely modulates pain through the inhibition of prostaglandin synthesis and the activity of nociceptive mediators. Flavonoids and tannins are believed to contribute to this antinociceptive activity.

We acknowledge that the present study focused on preliminary phytochemical screening and evaluation of the pharmacological activities of the methanolic leaf extract of *W*. *montana*, and as such, advanced phytochemical profiling—such as GC–MS or LC–MS analysis—was not conducted. Consequently, the specific bioactive constituents underlying the observed effects remain unidentified. This represents a key limitation of our current work.

## 5. Conclusion

This study underscores the broad‐spectrum pharmacological profile of the methanolic extract of the *W. montana* leaves, revealing significant antioxidant, anthelmintic, thrombolytic, cytotoxic, neuropharmacological, and antinociceptive activities—likely attributable to its rich array of bioactive phytoconstituents. Notably, the neuropharmacological effects indicate potential utility in managing neurological and mood‐related disorders, supporting its traditional use in central nervous system ailments. However, to translate these promising findings into therapeutic applications, further research is essential—particularly in elucidating the underlying molecular mechanisms, isolating and characterizing the active compounds, and conducting detailed preclinical safety and efficacy studies.

## Author Contributions

Millat Hossain Mesu: conceptualization, data curation, formal analysis, investigation, project administration, software, writing–original draft, and writing–review and editing. Md Ashraf Uddin Chowdhury and Mohammad Arman: conceptualization, investigation, methodology, project administration, resources, software, and writing–original draft. Israt Jahan and Sourav Kumar Shill: data curation, formal analysis, methodology, resources, validation, and visualization. Md. Al Mamun: formal analysis, investigation, resources, software, validation, visualization, and writing–original draft. Md. Jahirul Islam Mamun: data curation, methodology, resources, software, validation, and visualization. Md. Abdul Alim: investigation, resources, software, validation, visualization, and writing–original draft. Md. Tanvir Chowdhury: data curation, formal analysis, investigation, methodology, resources, and validation. S. M. Moazzem Hossen: conceptualization, formal analysis, investigation, project administration, resources, supervision, writing–original draft, and writing–review and editing.

## Funding

This study was not granted particular governmental, commercial, or nonprofit funding.

## Conflicts of Interest

The authors declare no conflicts of interest.

## Data Availability

The data that support the findings of this study are available from the corresponding author upon reasonable request.
